# Fertility intentions in the era of the new three-child policy in China: a cross-sectional survey of married adults of reproductive age

**DOI:** 10.3389/fpubh.2025.1674687

**Published:** 2025-11-14

**Authors:** Chenyun Zhang, Yingying Zhou, Yujing Ke, Wenchang Zhang, Mengqi Qin, Haridah Alias, Li Ping Wong

**Affiliations:** 1 School of Health Management, Fujian Medical University, Fuzhou, Fujian, China; 2 School of Public Health, Fujian Medical University, Fuzhou, Fujian, China; 3 The First Hospital of Lanzhou University, Lanzhou, Gansu, China; 4 Centre of Population Health (CePH), Department of Social and Preventive Medicine, Faculty of Medicine, Universiti Malaya, Kuala Lumpur, Malaysia; 5 Department of Medicine, College of Medicine, Korea University, Seoul, Republic of Korea

**Keywords:** three-child policy, fertility intention, married people, RMNCH support and services, cross-sectional study

## Abstract

**Background:**

China’s fertility rate has declined to an estimated record low of 1.09 in 2022, resulting in widespread broad negative impact on society and poses challenges for China’s economy. This study aimed to examine the fertility intentions of married adults under the new three-child policy and to investigate how demographic characteristics, support services, and perceived costliness and affordability of childbirth and childrearing influence these intentions.

**Methods:**

A large-scale web-based cross-sectional survey was carried out from 30 August 2021 to 1st May 2022. Study participants were married adults of reproductive age (18 to 49 years old) and Chinese citizens. The primary outcome was fertility intention. Factors associated with fertility intention namely (1) satisfaction with reproductive, maternal, newborn, and child health (RMNCH) services or support, and (2) affordability in childbirth and childrearing were collected.

**Results:**

A total of 2,996 complete responses were received in the survey. The desire for ≥ 3 children was 11.2%. Participants from the eastern region recorded the lowest proportion of desire for ≥ 3 children (8.2%). By demographics, males, lower educational achievement, sub-urban residency were significantly associated with higher intention to have ≥ 3 children. The association between perceived costliness of childbirth and childrearing cost and intention to have ≥ 3 children was not significant. A lower level of perceived affordability was significantly associated with a higher intention to have ≥ 3 children [adjusted odds ratio (aOR) = 1.63, 95% confidence interval (CI) 1.15–2.30]. Satisfaction with RMNCH services or support has no significant influence on fertility intention.

**Conclusion:**

Findings revealed that higher educational qualifications and urbanization are associated with lower fertility intention. Cost and support services may not be the main reasons driving low fertility desire.

## Introduction

On 31 May 2021, in response to findings from China’s 2020 Seventh National Population Census, which reported only 12.0 million births (approximately 18% fewer than in 2019) and a total fertility rate of 1.3, the government announced a universal three child policy and later amended regulations to allow couples to have up to three children (1–3). The census revealed China’s total fertility rate has declined from 1.6 live births per woman in 2017 to 1.3 in 2020 (4), which is on par with Japan (5). The falling fertility rate can have a broad negative impact on society and poses challenges for countries’ economies. With a low fertility rate, population aging will be rapid, hence shrinking the working-age population and potentially hampering economic growth and straining social and medical services (6, 7). In October 2015, the country decided to end its decades-long policy, and a two-child policy was introduced (8). During the two-child policy era, it was reported that the majority of women in China desired one or two children, while women in large cities stated a preference for only one child (9). There have been few reports noting reasons women desire fewer number of children. High child-rearing costs, particularly for child education was one of the most prominent reasons (9). Effects on parental lifestyle changes and the mother’s career development were reported to be also among the reasons women choose to have fewer children or decide not to have children at all (9).

Along with the current announcement of the new three-child policy, the authorities in China are also working to enhance supportive measures to facilitate couples in various aspects of childbearing, childcare, parenting, education, taxation, housing, and women’s rights in employment to encourage births (10). In recent years, many efforts have been designed to improve the Reproductive, Maternal, Newborn, and Child Health (RMNCH) in the community (11). Weaknesses in RMNCH delivery platforms, including limited access to care, and poor quality of services, among others, are major barriers to improving RMNCH outcomes (12). Ideally, strengthening the RMNCH services provision may promote satisfaction, and therefore enhance fertility intention. International evidence further supports this linkage. In Ethiopia, for example, women who received integrated maternal and child health services, including antenatal care, skilled birth attendance, and postnatal follow-up, were more likely to plan future pregnancies, indicating a positive link between RMNCH service utilization and fertility intentions (13). In Saudi Arabia, enhanced awareness of fertility-related issues, including age-related decline and broader reproductive health, is associated with more defined childbearing plans among educated young women, suggesting that improved reproductive health information and service integration could positively shape fertility intentions (14).

Beyond health system influences, cross-national evidence shows that fertility intentions are also shaped by broader demographic, economic, and cultural factors. Declining fertility is strongly linked to urbanization, delayed childbearing, and women’s labor force participation (15). Economic pressures, including housing costs, employment insecurity, and the opportunity costs of childrearing, further widen the gap between desired and achieved fertility (16, 17). Despite these international findings, existing studies often lack a critical synthesis of how demographic, economic, and RMNCH-related factors jointly shape fertility intentions, leaving important gaps that the present study seeks to address. This gap is particularly evident in the Chinese context, where most prior work has examined these dimensions in isolation, limiting a more comprehensive understanding of the interplay between service provision, affordability, and sociocultural context.

In light of the new reform in China, it is important to examine the extent to which the three-child policy is supported by married couples. Despite many testimonies that the costs of raising a child are a reason women decide to have more children (18, 19), empirical evidence for associations between fertility intention and parenting cost is lacking. Further, the satisfaction with the current RMNCH services provision has never been investigated in China. We hypothesized that better RMNCH services or support, coupled with perceived lower parenting cost, influence the extent to which individuals desire or intend to have a certain number of children. Henceforth, the aim of this study is to assess the fertility intention among married people in China and its influencing factors. The factors investigated were (1) demographics, (2) perceived costliness and affordability of childbirth and childrearing cost, and (3) perceived satisfaction with RMNCH services or support. This study may provide insight into critical elements of parenting costs and RMNCH services that affect people’s fertility intention and that should therefore be targeted specifically.

## Methodology

### Study participants and survey design

A large-scale cross-sectional survey using an online questionnaire was conducted from 30 August 2021 to 1st May 2022. The inclusion criteria were married adults of reproductive age (18 to 49 years of age), Chinese citizens, and able to comprehend and read Chinese. Including married adults as an inclusion criterion in the study is based on the understanding that married individuals are more likely to engage in family planning and childbearing considerations. This focus aligns with the goals of the new three-child policy, which primarily targets couples, especially those in stable relationships. Furthermore, societal norms in China typically associate childbearing with marriage, making married adults a vital demographic for assessing the policy’s impact.

The exclusion criteria included individuals who were unable to comprehend or read Chinese, and those with medical conditions that significantly impair fertility. Data collection was carried out in six regions in mainland China; North, Northeast, East, South central, Southwest and Northwest regions. A non-probability sampling approach was adopted using WeChat (the most popular message app in China) due to feasibility and its wide coverage across China; while this may bias toward more urban users, additional outreach via a survey company was used to enhance regional diversity. The research team sent the survey link to their colleagues and students, who are dispersed throughout the country, encouraging them to circulate and promote the link within their networks. A convenience sampling method was used in data collection. Specifically, we used WeChat Moments, a feature similar to a social media feed, to post engaging and informative content related to our survey. We crafted message that highlights the importance and relevance of the survey, along with a call-to-action prompting users to participate by clicking on the provided survey link. We also identified relevant WeChat groups or communities that align with the target audience of our survey and sought permission from the group administrators to share the survey link with their members. To ensure representation from people across all regions in China, a data collection company, unrelated to the researchers, was also hired. The company employed strategies such as targeted advertisements and promotions on WeChat, to reach participants from diverse geographical locations across China (Appendix 1).

The sample size was calculated for each region using the formula: n = Z^2^ *P * (1-P)/d^2^ (20). A conservative prevalence of 50% was assumed, as this value maximizes variance when the true prevalence is unknown and therefore yields the largest required sample size, ensuring adequate power and precision. A margin of error of 0.05 (5%), and a 95% confidence level. The calculated sample size was 384 for each region (n = [1.96^2^ *0.5*(1–0.5)]/0.05^2^).

### Instruments

The questionnaire (Appendix 2) consisted of four parts: (1) demographic, (2) perceived costliness and affordability of childbirth and childrearing cost, (3) perceived satisfaction with RMNCH services or support, and (4) fertility intention.

#### Demographics background

Personal details collected include age, gender, ethnicity, highest educational level, monthly household income, locality, and current residing region. The personal details collected, such as age, gender, ethnicity, highest educational level, monthly household income, locality, and current residing region, were determined to provide essential demographic information about the participants. These indicators help researchers understand the characteristics of the individuals participating in the study, allowing for the analysis of how different factors may influence fertility intention. Age, gender, and ethnicity can provide insights into the diversity of the sample population, while educational level and income can offer information on socioeconomic status and educational background. Locality and current residing region help in understanding geographical variations and potential regional influences on fertility intention.

#### Perceived costliness and affordability in childbirth and childrearing cost

A total of 11 items regarding the perceived costliness and affordability of preconception care, childbirth, bringing up children, and children’s education were queried. Each item question was assessed in terms of perceived costliness and affordability. Costliness and affordability were conceptualized as distinct constructs. Costliness reflects how expensive an item is perceived to be, while affordability captures the family’s perceived capability to pay for it. Although related, these represent different dimensions of financial perception, and thus were measured and scored separately. Options answers for perceived costliness were on a 4-point Likert scale, with the items scored as 1 (not at all costly), 2 (slightly costly), 3 (very costly), or 4 (extremely costly). Options answers for perceived affordability were also on a 4-point Likert scale, with the items scored as 1 (very unaffordable), 2 (unaffordable), 3 (affordable), or 4 (highly affordable). The possible total score for perceived costliness and affordability ranged from 11 to 44, with higher scores representing greater levels of perceived costliness and affordability. The perceived costliness (Cronbach alpha, *α* = 0.893) and affordability (Cronbach alpha, *α* = 0.901) in childbirth and childrearing costs had acceptable internal consistency.

#### Satisfaction with the reproductive, maternal, newborn, and child health (RMNCH) support or services

Since no standardized questionnaire exists to specifically measure RMNCH satisfaction in the Chinese context, a self-developed instrument was used. This questionnaire assessed participants’ perceived satisfaction with RMNCH support and services, focusing on how well these services addressed local needs and suitability within their communities in China. The question items were developed based on the Chinese government guidelines on the promotion of the development of care services for infants and young children under the age of three by the State Council (Office of the State Council, 2019) (11) and the recent guidelines on optimizing fertility policies and promoting long-term balanced population development by the Communist Party of China (CPC) Central Committee and State Council (21). Participants were given “very unsatisfied” “unsatisfied” “satisfied” or “very satisfied” response options to the 13 items. The score of each option answer was assigned as 1, 2, 3, and 4, respectively. The total score ranged from 13 to 52, with a higher score indicating a higher level of satisfaction. The satisfaction with RMNCH scale in this study had acceptable internal consistency (Cronbach alpha, *α* = 0.929).

#### Fertility intention

Participants were queried about their intentions regarding having children. The question was phrased as: “How many children do you want altogether?” Option answers were, “*I do not intend to have any children*,” “*1 child*,” “*2 children*” “*3 children*” and “*more than 3 children*.”

The questions were developed by researchers and professionals with expertise in child and infant care and population development policies. The questionnaire was validated by a panel of experts who are familiar with the construct of interest. A pilot survey was conducted on a small sample of the intended respondents before executing a large-scale survey.

### Statistical analysis

We ran univariate analyses followed by a multilevel logistic regression with region specified as a clustering variable, including all factors (demographics, satisfaction, and affordability) showing significance (*p* < 0.05), to determine factors associated with the intention to have ≥ 3 children. The main outcome was dichotomized as “≥3 children” versus “other options” (none, one child, two children). This categorization was chosen to align with the policy’s explicit focus on whether couples intend to have exactly three children, consistent with the objectives of China’s newly introduced three-child policy. Odds ratios (OR), 95% confidence intervals (95% CI) and *p*-values were calculated for each independent variable. The intraclass correlation coefficient (ICC) was also calculated to quantify the proportion of total variance in fertility intention attributable to between-region differences. The ICCs below 0.01 are considered negligible for clustering effects (22). All analyses were also conducted using SPSS version 22.0 (SPSS Inc., Chicago, IL, USA). A *p*-value of less than 0.05 was considered statistically significant.

### Ethical consideration

This study was approved by the Fujian Medical University Research Ethics Committee, China (Approval: FJMU 2021 NO.136). The participants were informed that their participation was voluntary. Informed Consent was obtained by providing the consent document on the first page of the survey and requiring participants to click a button on the statement of agreement indicating consent to participation before proceeding with answering the survey. The ethical committee approved the online consent in the web-based survey. The survey did not collect any identifying information from participants when they completed the survey.

## Results

**Figure 1:**
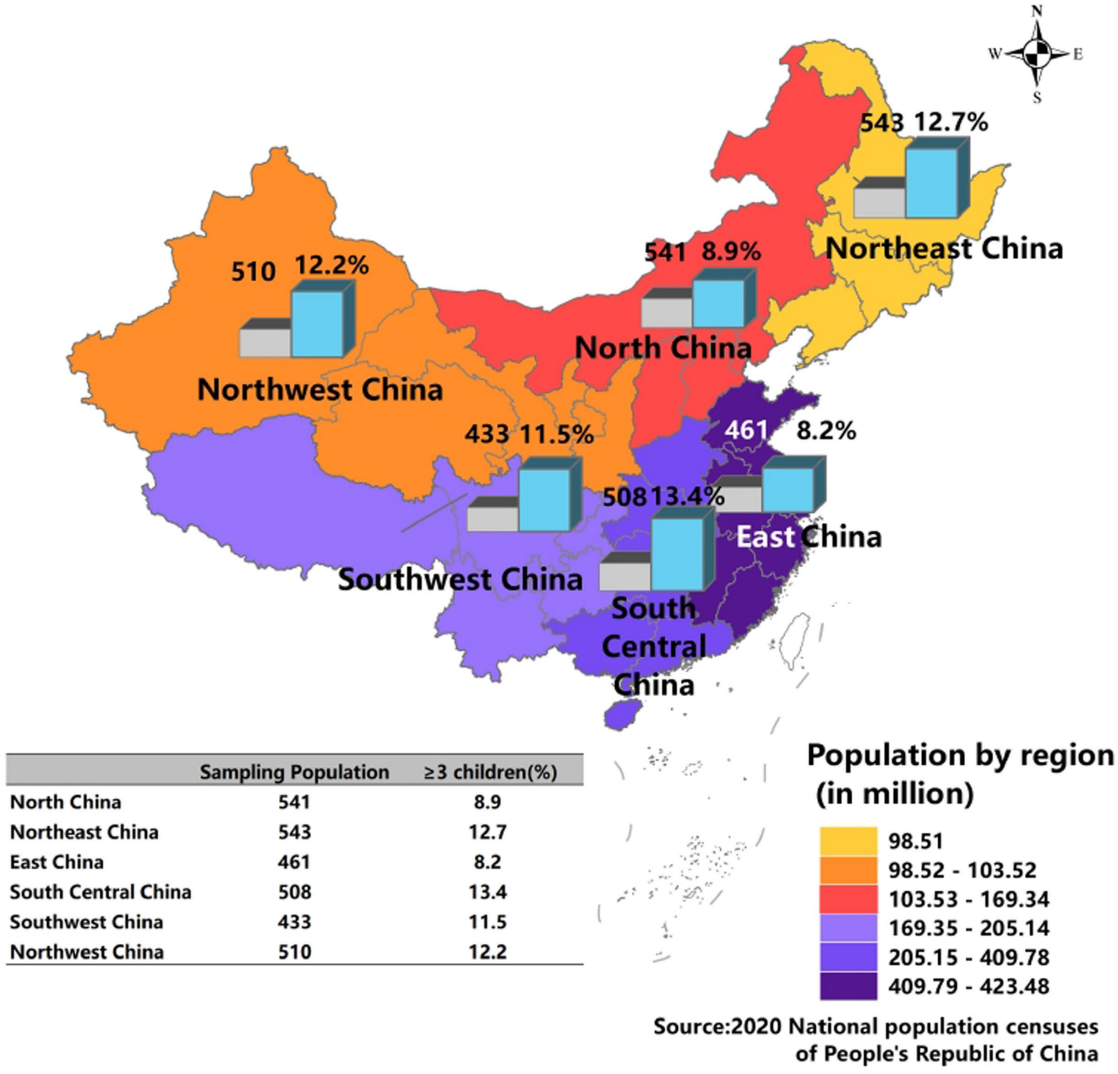
Proportion of responses and fertility intention by region.

A total of 2,996 complete responses were analyzed in the study. Figure 1 shows the distribution of responses by region. Most of the regions have almost equal responses, with Southwestern (*n* = 433) and Eastern (*n* = 461) regions recording slightly lower responses. Table 1 shows the demographics of the study participants. The age of the participants ranged from 18 to 49 years [mean = 31.7, standard deviation (SD) ± 5.5]. The majority were between 30–39 (57.3%) years old. There were almost equal proportions of male (44.9%) and female (55.1%) respondents. By education level, 25.1% had high school as the highest education level, while university graduates comprised 57.6%. Based on the income categories, a slightly low proportion reported an average monthly income of ≥ 15,000 CNY (28.6%). Most participants were from urban areas (58.9%) and sub-urban areas (26.9%).

**Table 1:** Demographic characteristics and factors associated with fertility intention using multilevel logistic regression with region specified as a clustering variable (*N* = 2,996).

	*N* (%)	Univariable analysis			Multivariable analysis	
		Do not want children/desire for 1 or 2 children (*n* = 2,661)	3 children and more (*n* = 335)	*p*-value^††^	≥ 3 children *vs*. do not want children/desire for 1 or 2 children OR (95% CI)	≥ 3 children *vs*. do not want children/desire for 1 or 2 children aOR (95% CI)^†^
Socio-demographic characteristics						
Gender						
Male	1,344 (44.9)	1,136 (84.5)	208 (15.5)	*p* < 0.001	2.19 (1.74–2.77)***	1.77 (1.40–2.24)***
Female	1,652 (55.1)	1,525 (92.3)	127 (7.7)		Reference	Reference
Age group (years)						
18–29	1,038 (34.6)	907 (87.4)	131 (12.6)	0.029	Reference	Reference
30–39	1717 (57.3)	1,547 (90.1)	170 (9.9)		0.77 (0.60–0.98)*	0.88 (0.69–1.13)
40–49	241 (8.0)	207 (85.9)	34 (14.1)		1.20 (0.79–1.82)	1.24 (0.82–1.87)
Highest educational level						
Secondary and below	518 (17.3)	417 (80.5)	101 (19.5)	*p* < 0.001	2.92 (2.21–3.88)***	1.79 (1.33–2.42)***
High school/Junior college	752 (25.1)	649 (86.3)	103 (13.7)		1.92 (1.46–2.53)***	1.45 (1.10–1.92)**
University	1726 (57.6)	1,595 (92.4)	131 (7.6)		Reference	Reference
Total monthly household income (CNY)						
9,999 and below	1,032 (34.4)	910 (88.2)	122 (11.8)	0.723	1.09 (0.82–1.45)	
10,000–14,999	1,106 (36.9)	986 (89.2)	120 (10.8)		1.00 (0.75–1.33)	
15,000 and above	858 (28.6)	765 (89.2)	93 (10.8)		Reference	
Residing location						
Urban	1764 (58.9)	1,627 (92.2)	137 (7.8)	*p* < 0.001	Reference	Reference
Sub-urban	807 (26.9)	661 (81.9)	146 (18.1)		2.60 (2.02–3.34)***	1.90 (1.46–2.47)***
Rural	425 (14.2)	373 (87.8)	52 (12.2)		1.66 (1.18–2.32)**	1.29 (0.91–1.83)
Perceived expensiveness and affordability of childbirth and childrearing cost						
Total perceived expensiveness of childbirth and childrearing cost score						
Low score (11–23)	1,396 (46.5)	1,287 (92.2)	109 (7.8)	*p* < 0.001	0.52 (0.41–0.66)***	0.95 (0.67–1.35)
High score (24–44)	1,600 (53.4)	1,374 (85.9)	226 (14.1)		Reference	Reference
Total perceived affordability of childbirth and childrearing score						
Low score (11–30)	1,381 (46.1)	1,163 (84.2)	218 (15.8)	*p* < 0.001	2.38 (1.88–3.02)***	1.63 (1.15–2.30)**
High score (31–44)	1,615 (53.9)	1,498 (92.8)	117 (7.2)		Reference	Reference
Perceived satisfaction of RMNCH services or support						
Total perceived satisfaction of RMNCH services or support score						
Low score (13–38)	1,491 (49.8)	1,291 (86.6)	200 (13.4)	*p* < 0.001	1.56 (1.24–1.97)***	0.97 (0.74–1.28)
High score (39–52)	1,505 (50.2)	1,370 (91.0)	135 (9.0)		Reference	Reference

^††^Statistical significance using Pearson’s chi-squared test.^†^Hosmer–Lemeshow test, Chi square: 5.899, *p*-value: 0.659; Nagelkerke R2: 0.098.*p < 0.05, **p < 0.01, ***p < 0.001.

**Figure 2:**
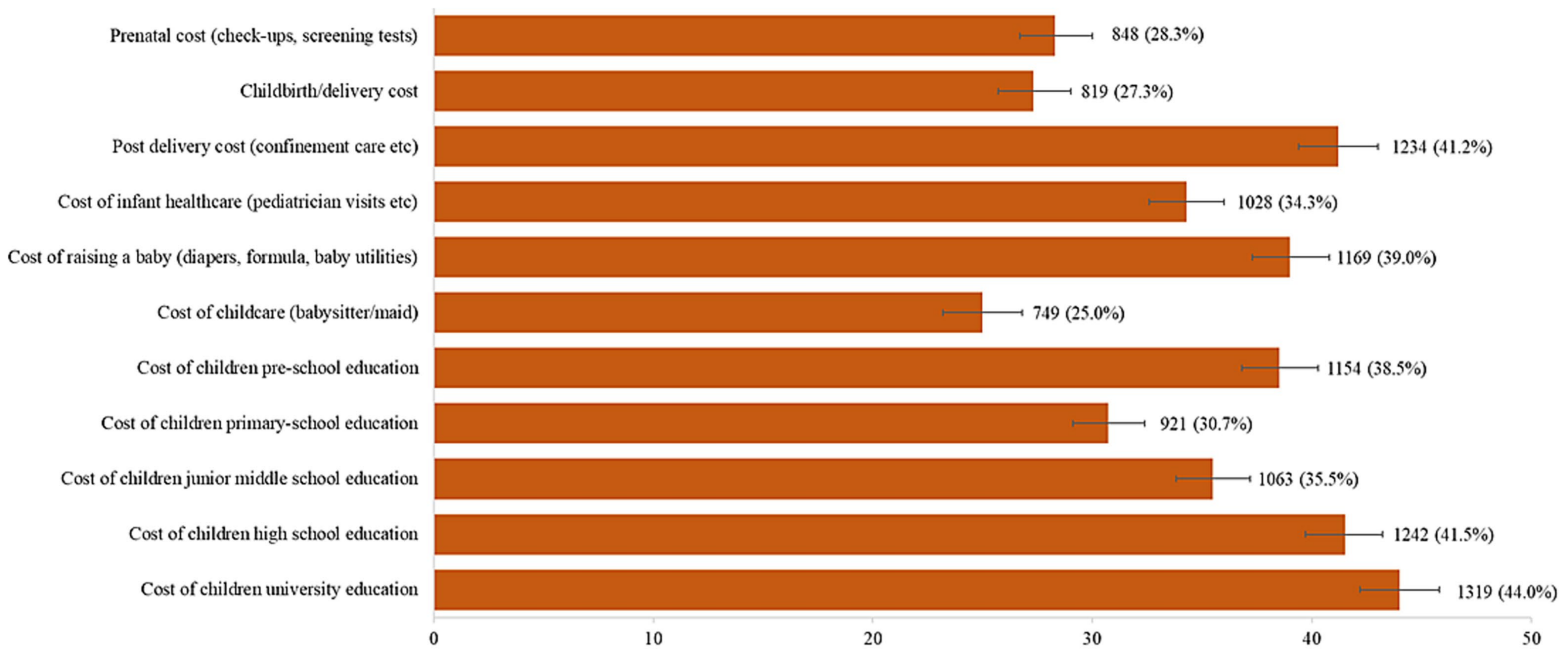
Proportion of participants who perceived childbirth and childrearing cost as *very costly/extremely costly* (*N* = 2,996).

Figure 2 shows the proportion of responses for individual items related to the perceived expensive childbirth and childrearing cost. The highest proportion (44.0%) reported cost of children university education as *very costly/extremely costly*. Cost of high school education (41.5%) and post-delivery cost (41.2%) accounted for the second and third highest proportion that rated as *very costly/extremely costly*. The total score of perceived expensive childbirth and childrearing cost ranges from 11 to 44, and the median was 24.0 (IQR 20.0–29.0). The total score of perceived expensive childbirth and childrearing cost was categorized as 11–23 or 24–44, based on the median split; as such, a total of 1,396 (46.5, 95% CI 44.8–48.4) were categorized as having a score of 11–23 and 1,600 (53.4, 95% CI 51.6–55.2) were categorized as having a score of 24–44 (Table 1).

**Figure 3:**
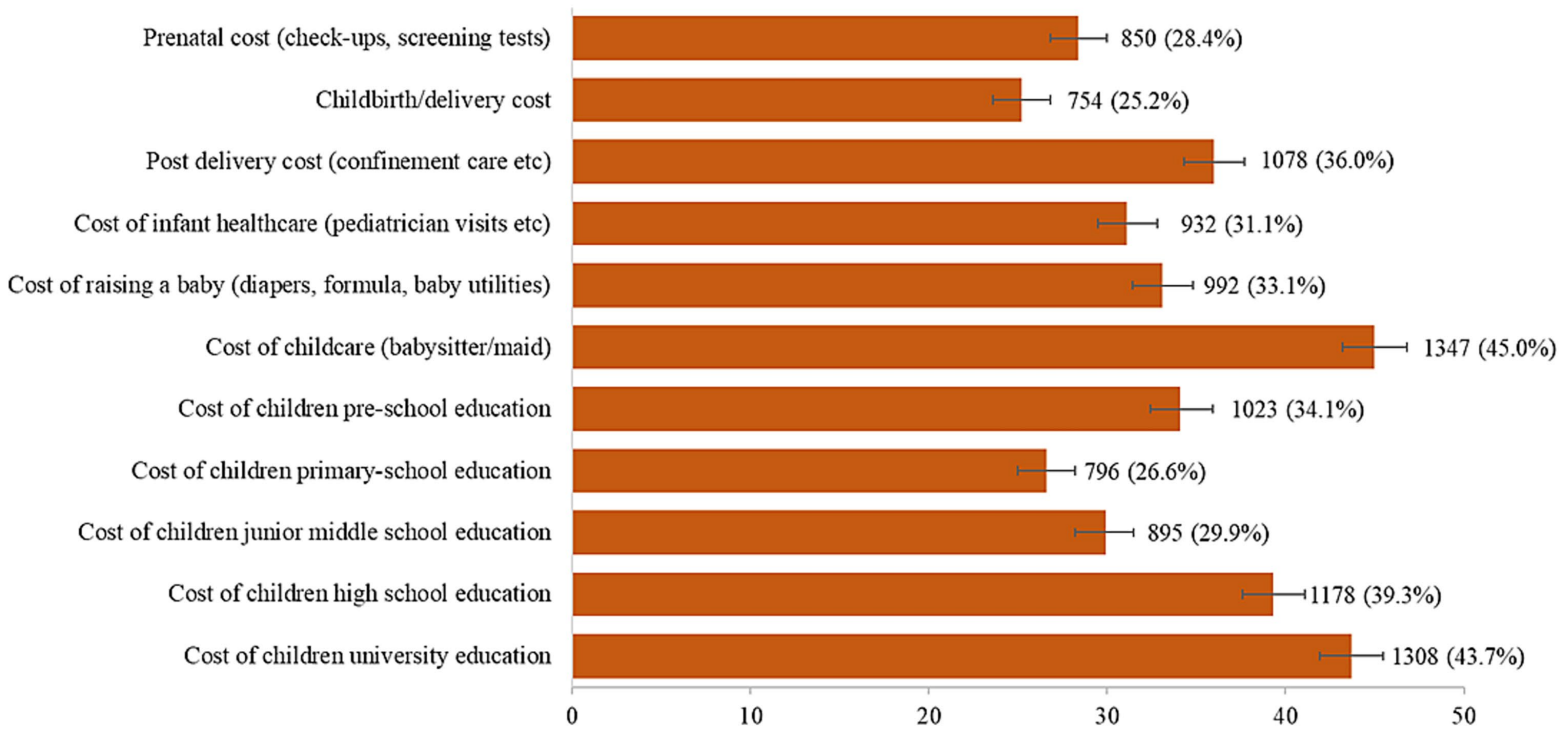
Proportion of participants who perceived affordability of childbirth and childrearing cost as *very unaffordable/unaffordable* (*N* = 2,996).

The proportion of responses on individual items of perceived affordability of childbirth and childrearing cost is shown in Figure 3. The highest proportion reported the cost of childcare (45.0%) as *very unaffordable/unaffordable*, followed by the cost of children’s university education (43.7%) and cost of children’s high school education (39.3%). The total score of perceived affordability of childbirth and childrearing costs ranges from 11 to 44, and the median was 31.0 (IQR 27.0–36.0). The total score of perceived affordability of childbirth and childrearing cost was categorized as 11–30 or 31–44, based on the median split; as such, a total of 1,381 (46.1, 95% CI 44.3–47.9) were categorized as having a score of 11–30 and 1,615 (53.9, 95% CI 52.1–55.7) were categorized as having a score of 31–44 (Table 1).

**Figure 4:**
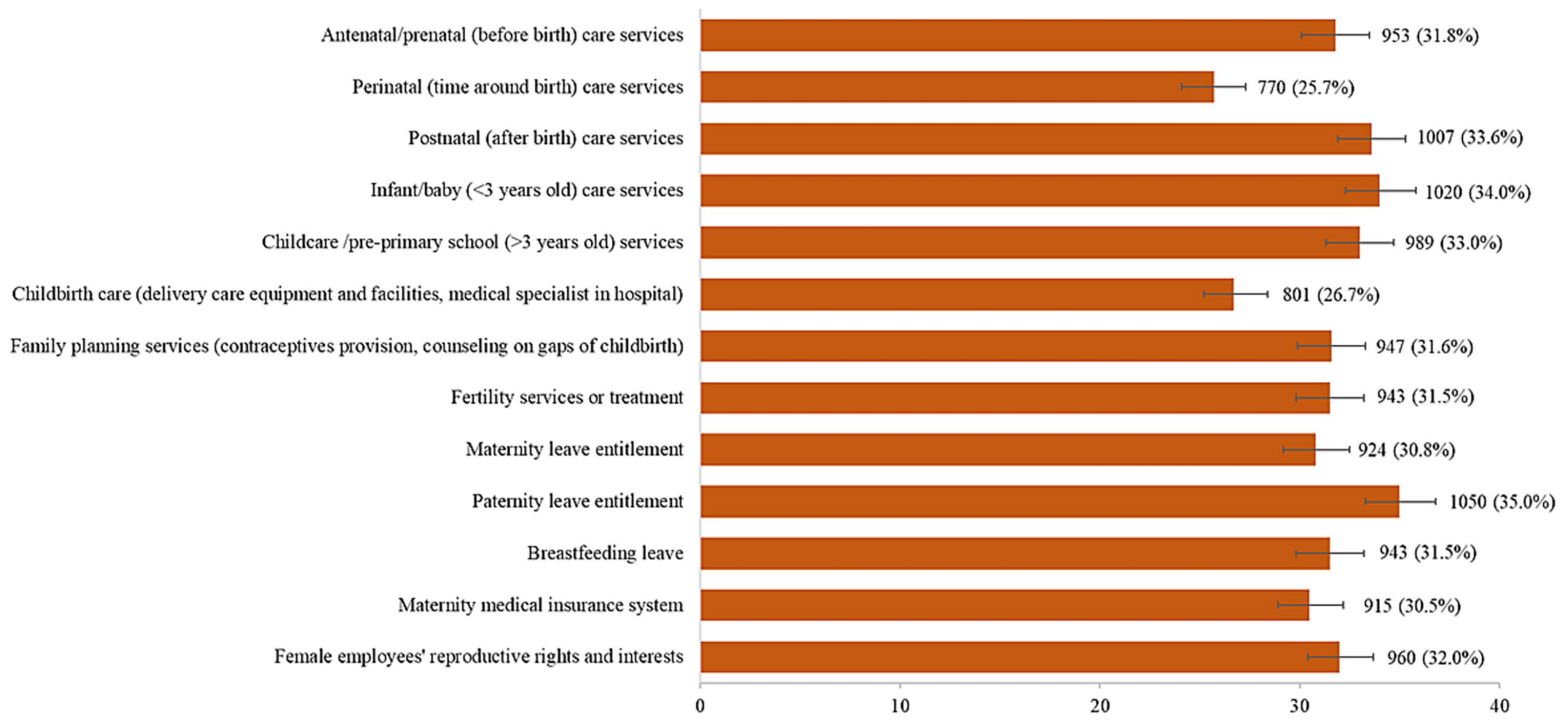
Proportion of participants who perceived satisfaction of RMNCH services or support as *very unsatisfied/unsatisfied* (*N* = 2,996).

Figure 4 shows the proportion of participants who perceived satisfaction with RMNCH services or support and fertility intention as *very unsatisfied/unsatisfied*. The highest proportion reported *very unsatisfied/unsatisfied* with paternity leave entitlement (35.0%), followed by infants <3 years care services (34.0%). The total score of satisfaction with RMNCH services or support ranges from 13 to 52, and the median was 39.0 (IQR 31.0–44.0). The total score of satisfaction with RMNCH services or support was categorized as 13–38 or 39– 52, based on the median split; as such, a total of 1,491 (49.8, 95% CI 48.0–51.6) were categorized as having a score of 13–38 and 1,505 (50.2, 95% CI 48.4–52.0) were categorized as having a score of 39–52.

Overall, a total of 10.1% (*n* = 304) reported an intent to have 3 children. In total, 11.2% (*n* = 335) reported an intent to have ≥ 3 children. The majority desired two children (50.6%, *n* = 1,517) (Appendix 3). As shown in Figure 1, the proportion that intends to have ≥ 3 children by region ranges from 8.2% (Eastern) to 13.4% (Southern Central). Table 1 shows the univariate and multivariable factors influencing fertility intention using multilevel logistic regression with region specified as a clustering variable. In the multivariable model, by demographics, males reported significantly higher intention to have ≥ 3 children than females (aOR = 1.77, 95% CI 1.40–2.24). There was a gradual decrease in intention to have ≥ 3 children by education level; participants with secondary school and below (aOR = 1.79, 95% CI 1.33–2.42) and high school (aOR = 1.45, 95% CI 1.10–1.92) exhibited significantly higher odds of intention to have ≥ 3 children than those with university education. There were no significant differences in the proportion to have ≥ 3 by household income and age groups. Participants residing in sub-urban (aOR = 1.90, 95% CI 1.46–2.47) expressed significantly higher intention to have ≥ 3 children than those in urban locations. The ICCs were below 0.01, indicating negligible clustering effects by region. The low ICC confirms that regional context contributed little and that the associations remained robust across regions.

There was no significant difference between the perceived costliness of childbirth and childrearing cost and intention to have ≥ 3 children. Participants with perceived affordability of childbirth and childrearing scores of 11–30 were found to have a significantly higher intention to have ≥ 3 children (aOR = 1.63, 95% CI 1.15–2.30) than those with a score of 31–44. There was no significant difference between perceived satisfaction with RMNCH services or support and intention to have ≥ 3 children.

## Discussion

### Overall fertility intentions and regional variations in fertility intentions

The profound shift in China’s population control program, allowing couples to have three children to achieve its intended goal of boosting population growth, received poor support from married adults in this study. Our findings indicate a limited level of support for larger families, with relatively few participants expressing an intention to have three or more children. Our findings align with the other two published studies conducted in China around the same period, which reported that 13% (23) and 15% (24) of their study population had an intention to have a third child, respectively. A publication that draws data from the National Fertility Survey conducted in 2017 found that 5% of women intended to have a third child (25). Of important note, our study was conducted in 2021 and 2022, following the subsiding of the COVID-19 pandemic. Although our survey did not include specific questions related to the pandemic, a potential period effect may have arisen during the 8-month data collection (late 2021–early 2022), as evolving post-COVID dynamics and localized outbreaks could have introduced short-term uncertainty that influenced fertility intentions and perceptions of affordability/RMNCH service satisfaction. Several studies have reported that the pandemic led to widespread uncertainty, economic difficulties, and health concerns, all of which could have influenced family planning decisions (26, 27), including the intention to have a third child in China (28, 29). However, when comparing data from the 2017 National Fertility Survey, which reported that 5% of women intended to have a third child, with more recent reports from our study and others (23, 24) conducted in 2021 and 2022, showing intentions ranging from 10 to 15%, it appears that the pandemic did not have a significant impact on the desire to have larger families. This rise in intention to have a third child, despite the uncertainties brought on by the pandemic, may perhaps suggest that the pandemic’s impact on fertility decisions was temporary or short-lived. As the pandemic subsided, people may have resumed their pre-pandemic family planning goals or even reassessed their priorities, leading to an increase in the intention to have more children. This upward trend in fertility intentions post-pandemic could also reflect a broader societal adjustment to the “new normal,” where concerns related to economic stability, health, and social circumstances began to ease, allowing couples to feel more secure in planning larger families. Additionally, it may indicate the effectiveness of government policies promoting larger families, which may have gained more traction as pandemic-related fears diminished.

Our study found approximately 50% intended to have two children, which was slightly lower compared to 60% in a recently published study (23). The results of this study confirm the reported results of previous studies in China (23, 24), suggesting that the relaxation in birth policy is not well supported by the public. We cannot, however, rule out the possibility that some people may support the three-child even though they do not wish to have a third child. The government campaigns may therefore be needed to enhance public support for the new three-child policy and public fertility intentions.

The current study found low desire for three children in the Eastern region, the economic center of China, highlighting potential regional disparities in demographic trends and population within China in the near future. This finding also holds significant ramifications in terms of demographics and economics in the Eastern region.

In Eastern China, lower fertility intentions can be partly explained by rapid urbanization and modernization, which tend to delay marriage and childbearing and reduce fertility desires. Studies have shown that in more urbanized provinces, individuals are more likely to postpone marriage or remain unmarried for longer periods, contributing to reduced fertility (30). Moreover, in economically developed areas such as Eastern China, women often attain higher levels of education and have greater employment opportunities, which are associated with delayed childbearing and preferences for smaller family sizes (31). In China, traditional values and cultural practices often revolve around larger families and strong kinship networks. The disconnection from kinship ties, especially among youth in urban areas, marks a profound transformation in Chinese society and culture, impacting the traditional collective family structure and practices (32).

Social interactions, community cohesion, and social ties play a vital role in fostering well-being, resilience, resource sharing, collaboration, and a higher quality of life in the context of a fast-paced and stressful city environment (33). Smaller family sizes can impact social interactions and community cohesion, resulting in a reduced sense of community and may potentially weaken social ties in the fast-paced and stressful city environment. These findings suggest the need for regionally differentiated strategies. In areas such as Eastern China, where fertility intentions are especially low, targeted policies could include housing incentives for young couples, regional subsidies for childrearing, and campaigns that promote work–family balance. At the national level, awareness initiatives could also emphasize the social and cultural value of family building, beyond purely economic considerations.

### Demographic disparities in fertility intention

This study also shows interesting demographic disparities in fertility intention. Firstly, the desire to have three children or more among males is higher than in females. In this study, the significance of gender in the multivariate model suggests that gender has an independent effect on fertility intention, with males showing higher odds of intending to have more than three children. This implies that gender is one of the key factors influencing fertility intentions, and males are more likely than females to express a desire for larger family sizes, regardless of other factors included in the model. Cross-cultural comparisons of gender differences in desired fertility across societies have long indicated higher fertility intention in males compared to females (34). Therefore, there is a need to work towards transforming traditional gender norms and stereotypes that influence fertility intentions. Promoting gender equality and empowering women to make autonomous decisions regarding family size is beneficial. Increasing education and awareness about family planning options, and the benefits of gender-equitable decision-making in family planning is warranted. Married couples in China should be encouraged to engage in open discussions about fertility intentions.

Secondly, this study evident the negative effect of education on fertility intention. This is inconsistent with previous research on the causal effect of women’s education on the number of children in China, which found that each additional year of women’s education increased the probability of having at least one child by 3 percentage points and the probability of having two or more children by 4 percentage points (35). Numerous previous reports (36) and a recent study of six global regions, including Asia, similarly reported that fertility rates correlate negatively with education (37). The dynamic interplay between education and fertility in China has been reported. It was theorized that an increase in educational attainment enhances their economic opportunities, leading to higher decision-making power in birth control, as well as having the desired number of children than their less-educated counterparts (38). The gender equity theory suggests a decline in fertility with women’s rising participation in education and employment, making women dually burdened with employment and care of their families. The theory implies that greater gender equity within the family, whereby men take equal responsibilities in family life with their partners, has a strong fertility-encouraging effect (39).

Thirdly, participants who lived in sub-urban areas had a higher likelihood of desiring more children, compared to urban dwellers. Urban–rural fertility variation in China (40) aligns with evidence from other countries (41, 42) that found that urban residence is associated with lower fertility desires. The impact of urbanization on fertility transitions warrants serious attention. Changes in the urban environment policies and interventions to increase fertility intentions of urban dwellers are desirable. Implementation of family-friendly policies and interventions in urban areas to address the challenges faced by couples in balancing work and family life is useful. This can include flexible work arrangements, affordable and accessible childcare services, and extended parental leave options. There is also a need to improve housing and urban planning. Considering the impact of housing affordability and fertility intentions (43), it is important to implement urban planning strategies that prioritize affordable housing for urban folks.

The current study however, revealed no specific trend between household income and fertility intention. The unclear pattern was similarly found in a recent study in Korea (44). Unlike in the past when an increase in income was generally recognized to increase the fertility rate, it was hypothesized that the increasing phenomenon of not favouring additional children with an increase in income observed today is caused by the shift in society and cultural attitudes in favor of smaller family sizes. Couples have a higher desire for personal fulfillment, career aspirations and quality of life than for additional children (44, 45). Therefore, addressing demographic disparities will require gender-sensitive and education-sensitive interventions. For example, workplace reforms that promote gender equality in caregiving, such as extended paid paternity leave, flexible work hours, and employer-supported childcare, could help reduce the burden on women with higher educational attainment. In rural and sub-urban areas, continued investment in healthcare access, education, and infrastructure may sustain fertility intentions, while in urban centers, more affordable housing and improved work–life balance policies are crucial.

### Costliness of childbirth and childrearing, and fertility intention

The study also did not find a significant association between the perceived costliness of childbirth and childrearing cost and fertility intention. In contrast, there was a significant inverse association between perceived affordability of childbirth and childrearing cost and fertility intention. Findings suggest that financial factors may not be entirely the main reason driving low fertility desire in this dataset. This counterintuitive finding suggests that even when financial constraints are absent, fertility preferences remain low, as desired family size is shaped by many factors beyond cost. The burden of raising children extends beyond money, including concerns that larger families may reduce living standards, the stress of parenting, and significant time sacrifices (46). These pressures are especially heavy for women, who face motherhood penalties in the labor market while carrying the main responsibility for housework and caregiving (47). A recently published study in China reported that the biggest barrier to rearing children aged 0–3 was time cost (39.3%), followed by the cost of childrearing (36.5%) and child education cost (13.5%) (24). This may suggest, within the limits of our cross-sectional design, that affordability was not a dominant concern for couples considering having more children.

Another explanation could be that increasingly young people in China increasingly have a different mindset than the older generation. In recent years, China has experienced significant cultural changes that impacted individuals’ fertility intentions (48). Marriage, family, and children, as well as the large family structure, may no longer hold the same value or priority. Thus, while affordability might still matter, the stronger influence of cultural norms, gender equity, and time-related opportunity costs of parenting appear to be reshaping fertility decisions. Although the rising cost of raising a child and the desire for personal freedom are often cited as the main reasons for having fewer or no children, our findings resonate with recent sociological evidence showing that cultural shifts and social norms play a significant role. For instance, Yu and Liang (48) demonstrated that neighborhood and group-level social norms strongly shape fertility intentions in China, underscoring how social context can constrain or enable reproductive choices. Similarly, Zhao et al. (49) reported that offline social capital tends to increase fertility intentions, whereas online social capital may reduce them, suggesting that evolving social interactions influence preferences for smaller families. This perhaps explains the earlier note about no specific trend between household income and fertility intention. Despite a high income, couples desire small families. Many prioritize career development, personal freedom, and the enjoyment of material wealth. Upholding traditions of their family culture and values is no longer important in modern life. Future research is needed to confirm this and to understand the strength of how this new cultural shift affects couples’ fertility intentions. Since affordability was not strongly associated with fertility intentions in this dataset, policy measures should move beyond direct financial subsidies. Instead, reforms could prioritize reducing time-related costs of parenting through expansion of high-quality, affordable childcare services, after-school programs, and flexible parental leave schemes. Introducing state-supported childcare vouchers and strengthening early childhood education could directly address practical barriers, particularly for dual-income households in urban areas.

### RMNCH services or support, and fertility intention

Although this study found no significant association between fertility intention and RMNCH support or services, a considerable high proportion reported dissatisfaction with paternity leave and infant care services. Similarly found in another study, sufficient parental leave and available and qualified childcare services are among the suggested supporting measures that should be enhanced to improve fertility desire (24). Cultural and attitudinal dimensions also interact with service provision. Peng et al. (50) found that among Chinese married youth during COVID-19, parenting perceptions and social expectations shaped fertility intentions as much as material conditions, reinforcing the importance of considering cultural context when designing fertility-supportive policies.

Our current study sheds light on married couples’ fertility intention and their associated concerns. Nonetheless, the study has some limitations that should be acknowledged and considered. First, the use of an online survey may have resulted in sampling bias, so the results may not be generalisable to the wider community, as reflected in the lack of representation from some locations. Second, the issue of self-reporting bias may represent a potential problem in the validity of the assessment. Thirdly, period effects may have an unfavorable influence on our study. The prolonged duration of data collection (8 months) may have subjected respondents who completed the survey at different times to varying policies and socio-economic conditions, potentially influencing their attitudes and intentions. Additionally, it is important to note that the measurement indexes utilized in our study were developed internally, taking into account the Chinese government guidelines on the promotion of care services for infants and young children under the age of three, as outlined by the State Council and the guidelines on optimizing fertility policies and promoting long-term balanced population development by the Communist Party of China (CPC) Central Committee and State Council. Therefore, it is advisable to interpret the results with caution, considering the potential limitations of these self-developed measures. Strengthening RMNCH support systems should therefore focus on expanding parental leave entitlements for both mothers and fathers, integrating paternity leave as a standard benefit, and improving the availability of qualified infant care services, especially for children under 3 years of age. Enhanced workplace protections for parents, together with community-based support networks, may help to create an environment more conducive to larger family sizes.

### Limitations

Several potential limitations in our study warrant consideration. First, the cross-sectional design restricts our ability to draw causal inferences, and all observed relationships should be interpreted as associations rather than causal effects. Secondly, the use of an open, online survey design, which may introduce selection bias due to self-selection, impacting the generalizability of the results. Additionally, the system’s inability to capture response rates hinders the ability to assess the representativeness of the sample. This limitation may result in the underrepresentation of certain demographic groups. Another limitation of this study is the use of convenience sampling and the absence of regional stratified sampling. As recruitment was conducted primarily through WeChat networks, there is a risk of over-representation of urban and more educated participants, while rural groups may have been under-represented. Given that fertility intentions may differ substantially across provinces and between urban, sub-urban, and rural settings, this imbalance could further limit the generalizability of our findings. As a result, while our big sample of 2,996 respondents provides valuable insights, it differs from national demographics. Participants in our study tended to have higher educational attainment and were more likely to reside in urban or suburban areas compared with the general population, while rural respondents were underrepresented. On income, 28.6% of our sample reported household income of at least CNY 15,000 per month, which indicates a relatively affluent sample compared with the 2023 national per capita disposable income of CNY 39,218 per year, with urban residents averaging CNY 51,821 and rural CNY 21,691 (51). These differences in education, urbanicity, and income may limit the generalizability of our findings to the broader Chinese population. Future research should consider using stratified sampling to better capture regional differences and enhance the accuracy of the results. Another limitation of this study is that we did not collect information on participants’ existing number of children, which is an important determinant of fertility intention. The absence of this variable may have introduced residual confounding, and future studies should incorporate parity to better adjust for potential confounders. It is also important to note that the personal details collected, including age, gender, ethnicity, highest educational level, monthly household income, locality, and current residing region, are determined based on the participants’ self-reported information provided during the survey. Therefore, the data may be subjected to self-report bias. Further limitation of our study is the exclusion of individuals who cannot read Chinese, which may have resulted in the underrepresentation of illiterate individuals in China. This may particularly affect people in rural or disadvantaged areas where literacy rates tend to be lower. However, it is important to note that the number of reproductive-age individuals who are illiterate or unable to read Chinese is relatively small in China today, and thus the overall impact on the study’s findings is likely minimal. Nonetheless, future studies should consider including illiterate populations to ensure a more comprehensive representation of the Chinese population.

It is also important to acknowledge several limitations related to the questionnaire tools used in this study. First, the use of a 4-point Likert scale without a neutral midpoint may have constrained participants’ ability to express ambivalence. Furthermore, the measurement of fertility intentions itself has been a longstanding challenge, with scholars emphasizing the importance of distinguishing between short-term versus long-term intentions, desired versus expected fertility, and accounting for the “intention–behavior gap” (52). Recent evidence also highlights the instability of fertility preferences over time, showing that while individuals often perceive their desires as stable, they are in fact highly context-dependent and subject to change (53). In our study, only general or unstipulated fertility intentions were measured, without differentiation across these critical dimensions, which should be acknowledged when interpreting the findings. In addition, although the self-developed RMNCH satisfaction and affordability instruments demonstrated high internal consistency and were grounded in national policy frameworks in China, their lack of prior validation and limited comparability with standardized international tools may constrain external validity. However, these instruments were derived from national policy guidelines, expert-reviewed, and showed acceptable internal consistency for exploratory research. Future studies should undertake psychometric validation and cross-cultural adaptation to strengthen the robustness of these measures for use across different countries.

The previous one-child and later two-child policies in China may have influenced respondents’ attitudes toward family planning, potentially introducing a bias known as acquiescence, where individuals conform to or align their responses with established state policies. For decades, these policies shaped societal norms and expectations around family size, particularly in urban areas where compliance was strictly enforced. As a result, many individuals may still hold residual preferences for smaller families, despite the new three-child policy. Future studies should investigate whether acquiescence to past policies is influencing respondents’ fertility intentions. Lastly, the long 8-month data collection period may have introduced a potential period effect, as evolving post-COVID conditions, policy changes, and social dynamics during this time could have influenced fertility intentions and perceptions of affordability and RMNCH service satisfaction. Despite all the noted limitations, this research is advantageous due to its nationwide scope and large sample size. It lays the groundwork for more comprehensive future studies and identifies new avenues for exploration.

### Recommendation for future research

Future research should focus on several key areas to better understand fertility intentions in China under the new three-child policy. Longitudinal studies are needed to track how fertility intentions evolve over time with changing socio-economic conditions. Additionally, investigating regional disparities, particularly in the economically significant Eastern region, will provide insights into how local conditions and cultural shifts affect family size preferences. Urbanization’s effect on fertility intentions should be closely examined, particularly concerning housing affordability, childcare access, and the effectiveness of family-friendly policies. Understanding how urban living conditions influence fertility desires can help in developing supportive policies for urban couples. Finally, future research should delve into the cultural shifts affecting fertility intentions. Investigating changing attitudes toward marriage, family, and children will help explain low fertility desires despite financial stability. Future studies should delve deeply into how perceived costs of childbirth and childrearing, as well as the availability of RMNCH services, influence fertility intentions. Addressing these areas will provide a comprehensive understanding of the factors influencing fertility intentions in China and inform policy development to support population growth.

## Conclusion

Despite easing birth restrictions aimed at boosting the country’s stagnating population growth, the majority of married people have low fertility intentions. This study revealed that couples embrace the concept that one or two children are enough, and a minority reported they are not interested in having children at all. Such social norm shifts may further drag down birth rates in China. Policymakers must take a concrete approach in designing interventions to address the demographics and regional inequalities in desired fertility. Full consideration should be given to urbanization’s effect on low fertility intention and specifically targeting the higher educated people to encourage them to have a second or third child. While financial affordability and fertility-related support services may no longer be the primary barriers for couples to have more children, they may still play a facilitating role. Therefore, it remains important to explore and identify other concrete factors beyond cost and service availability that contribute to low fertility intention. Our findings point to several actionable policy directions. Structural measures that reduce the time costs of parenting and address gender inequality may be more effective than generic financial incentives. Specific interventions could include: targeted housing incentives in urban centers to reduce the economic burden of family formation; parental leave reforms with stronger paternity leave provisions to promote gender equity in caregiving; childcare subsidies and vouchers to lower direct costs; and the expansion of high-quality infant care and early childhood education services to reduce the opportunity costs of childrearing. For rural and sub-urban areas, investments in healthcare access, education, and infrastructure may sustain existing fertility intentions, while urban-focused policies could help mitigate declining intentions in cities. Integrating these evidence-based strategies into fertility policy may create a more supportive environment for larger families in China. At the same time, the conclusions of this study should be interpreted with caution given its reliance on self-reported perceptions and sampling limitations. Future research employing more representative and longitudinal designs is necessary to confirm these findings and to better capture the complex determinants of fertility intentions over time.

## Data Availability

The datasets presented in this study can be found in online repositories. The names of the repository/repositories and accession number(s) can be found below: The anonymized datasets have been deposited in Figshare and are publicly available at https://doi.org/10.6084/m9.figshare.30134956.
