# Supplementary figures and images for: Fertility intentions in the era of the new three-child policy in China: a cross-sectional survey of married adults of reproductive age
Source: Front Public Health. 2025 Nov 14;13:1674687. doi: 10.3389/fpubh.2025.1674687 (PMC12660072; doi:10.3389/fpubh.2025.1674687)

Overall Male Female

Do not want any children

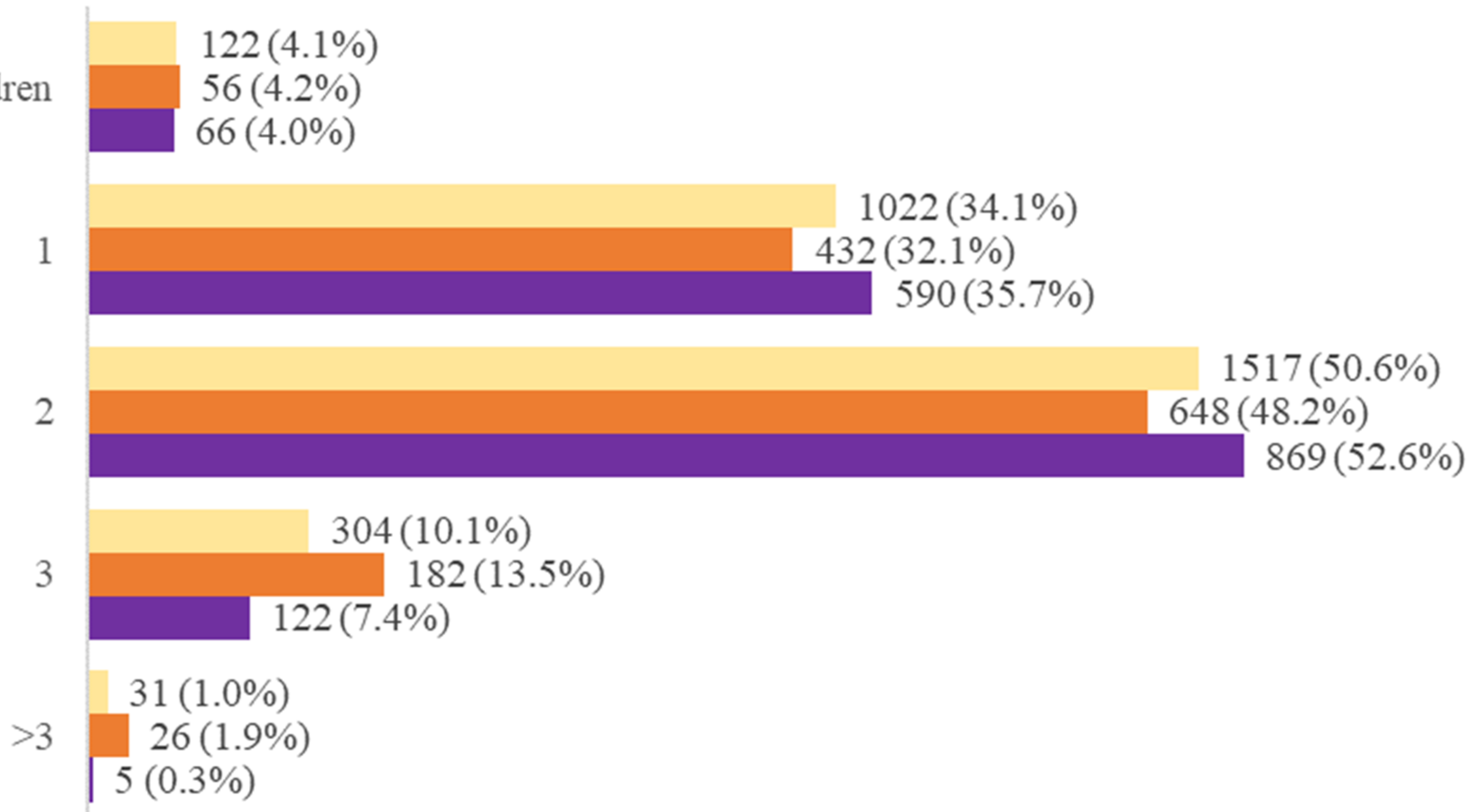

Supplement: Supplementary file 1 [file Data_Sheet_1.PDF]

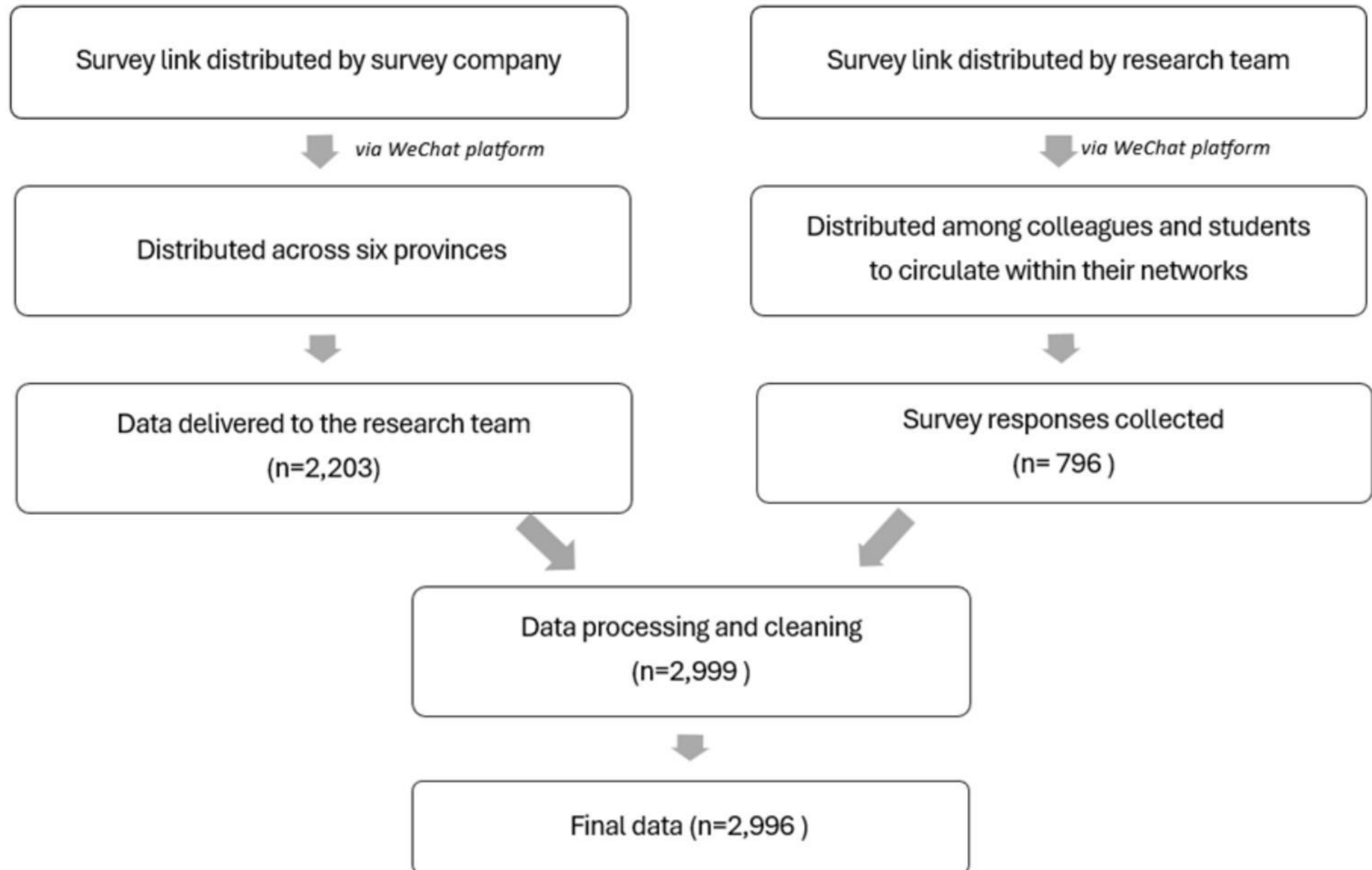

Supplement: Supplementary file 2 [file Data_Sheet_2.PDF]
