# Supplementary material for: Fertility intentions in the era of the new three-child policy in China: a cross-sectional survey of married adults of reproductive age
Source: Front Public Health. 2025 Nov 14;13:1674687. doi: 10.3389/fpubh.2025.1674687 (PMC12660072; doi:10.3389/fpubh.2025.1674687)
Supplement: Supplementary file 3 [file Data_Sheet_3.pdf]

A national survey of married people's views about China's three-child

Section A
GENERAL INFORMATION

**Demographic Background**		
A1	Sex	[1] Male<br>[2] Female
A2	Age	___ years old
A3	Ethnicity	[1] Han<br>[2] Others
A4	Highest education level	[1] No formal education<br>[2] Primary school<br>[3] Junior middle school<br>[4] Secondary specialized school<br>[5] High school<br>[6] Junior college<br>[7] University and above
A5	Total household income/Family monthly income in CNY	[1] <4000<br>[2] 4000-9999<br>[3] 10000-14999<br>[4] 15000-19999<br>[5] ≥20000
A6	Current location	[1] Urban<br>[2] Sub-urban<br>[3] Rural
A7	Current Residing Region	[1] North China<br>[2] Northeast China<br>[3] East China<br>[4] South Central China<br>[5] Southwest China<br>[6] Northwest China
**Family size preference**		
A8	How many children do you want all together?	[1] I don't want any children<br>[2] 1<br>[3] 2<br>[4] 3<br>[5] >3

[¶]Northern region (Beijing, Tianjian, Hebei, Shanxi, Inner Mongolia); Northeast China (Liaoning, Jilin, Heilongjiang); East region(Shanghai, Jiangsu, Zhejiang, Anhui, Fujian, Jiangxi, Shandong,); Southern

Central China (Hunan, Guangdong, Guangxi, Hainan, Henan, Hubei); Southwest China (Chongqing, Sichuan, Guizhou, Yunnan, Tibet); Northwest China (Shannxi, Gansu, Qinghai, Ningxia, Xinjiang).

Section B
Cost and Affordability

Perception of costliness and affordability in childbirth and childrearing cost

		1. Costliness	2. Affordability
	**The following are the cost of having a baby, from pregnancy to baby's first birthday**		
B1	Prenatal cost (check-ups, screening tests)	[1] Not at all costly<br>[2] Slightly costly<br>[3] Very Costly<br>[4] Extremely costly	[1] Highly affordable<br>[2] Affordable<br>[3] Unaffordable<br>[4] Very unaffordable
B2	Childbirth/delivery Cost	[1] Not at all costly<br>[2] Slightly costly<br>[3] Very Costly<br>[4] Extremely costly	[1] Highly affordable<br>[2] Affordable<br>[3] Unaffordable<br>[4] Very unaffordable
B3	Post delivery cost (confinement care etc.)	[1] Not at all costly<br>[2] Slightly costly<br>[3] Very Costly<br>[4] Extremely costly	[1] Highly affordable<br>[2] Affordable<br>[3] Unaffordable<br>[4] Very unaffordable
B4	Cost of infant healthcare (pediatrician visits, etc)	[1] Not at all costly<br>[2] Slightly costly<br>[3] Very Costly<br>[4] Extremely costly	[1] Highly affordable<br>[2] Affordable<br>[3] Unaffordable<br>[4] Very unaffordable
B5	Cost of raising a baby (diapers, formula, baby utilities)	[1] Not at all costly<br>[2] Slightly costly<br>[3] Very Costly<br>[4] Extremely costly	[1] Highly affordable<br>[2] Affordable<br>[3] Unaffordable<br>[4] Very unaffordable
B6	Cost of childcare (babysitter/maid)	[1] Not at all costly<br>[2] Slightly costly<br>[3] Very Costly<br>[4] Extremely costly	[1] Highly affordable<br>[2] Affordable<br>[3] Unaffordable<br>[4] Very unaffordable
**The following are the cost of children education**			
B7	Cost of children pre-school education	[1] Not at all costly<br>[2] Slightly costly<br>[3] Very Costly<br>[4] Extremely costly	[1] Highly affordable<br>[2] Affordable<br>[3] Unaffordable<br>[4] Very unaffordable
B8	Cost of children primary-school education	[1] Not at all costly<br>[2] Slightly costly<br>[3] Very Costly<br>[4] Extremely costly	[1] Highly affordable<br>[2] Affordable<br>[3] Unaffordable<br>[4] Very unaffordable

B9	Cost of children junior middle school education	[1] Not at all costly<br>[2] Slightly costly<br>[3] Very Costly<br>[4] Extremely costly	[1] Highly affordable<br>[2] Affordable<br>[3] Unaffordable<br>[4] Very unaffordable
B10	Cost of children high school education	[1] Not at all costly<br>[2] Slightly costly<br>[3] Very Costly<br>[4] Extremely costly	[1] Highly affordable<br>[2] Affordable<br>[3] Unaffordable<br>[4] Very unaffordable
B11	Cost of children university education	[1] Not at all costly<br>[2] Slightly costly<br>[3] Very Costly<br>[4] Extremely costly	[1] Highly affordable<br>[2] Affordable<br>[3] Unaffordable<br>[4] Very unaffordable

Section C
Perception of current services

**Please rate your level of satisfaction with the current services in your area**		**Very unsatisfied**	**Unsatisfied**	**Satisfied**	**Very satisfied**
C1	Antenatal/prenatal (before birth) care services	[1]	[2]	[3]	[4]
C2	Perinatal (time around birth) care services	[1]	[2]	[3]	[4]
C3	Postnatal (after birth) care services	[1]	[2]	[3]	[4]
C4	Infant/baby (<3 years old) care services	[1]	[2]	[3]	[4]
C5	Childcare /pre-primary school (>3 years old) services	[1]	[2]	[3]	[4]
C6	Childbirth care (delivery care equipment and facilities, medical specialist in hospital)	[1]	[2]	[3]	[4]
C7	Family planning services (contraceptives provision, counseling on gaps of childbirth)	[1]	[2]	[3]	[4]
C8	Fertility services or treatment	[1]	[2]	[3]	[4]
C9	Maternity leave entitlement	[1]	[2]	[3]	[4]
C10	Paternity leave entitlement	[1]	[2]	[3]	[4]
C11	Breastfeeding leave	[1]	[2]	[3]	[4]
C12	Maternity medical insurance system	[1]	[2]	[3]	[4]

C13	Female employees' reproductive rights and interests	[1]	[2]	[3]	[4]
